# Enhanced nanoparticle delivery exploiting tumour-responsive formulations

**DOI:** 10.1186/s12645-018-0044-6

**Published:** 2018-11-21

**Authors:** Lindsey A. Bennie, Helen O. McCarthy, Jonathan A. Coulter

**Affiliations:** School of Pharmacy, Queens University Belfast, Lisburn Road, Belfast, BT9 7BL UK

**Keywords:** Tumour microenvironment, Nano-therapeutics, Gold nanoparticles, Cell-penetrating peptide, Cleavable stealth molecules, Nucleic acids

## Abstract

Nanoparticles can be used as drug carriers, contrast agents and radiosensitisers for the treatment of cancer. Nanoparticles can either passively accumulate within tumour sites, or be conjugated with targeting ligands to actively enable tumour deposition. With respect to passive accumulation, particles < 150 nm accumulate with higher efficiency within the tumour microenvironment, a consequence of the enhanced permeability and retention effect. Despite these favourable properties, clinical translation of nano-therapeutics is inhibited due to poor in vivo stability, biodistribution and target cell internalisation. Nano-therapeutics can be modified to exploit features of the tumour microenvironment such as elevated hypoxia, increased pH and a compromised extracellular matrix. This is in contrast to cytotoxic chemotherapies which generally do not exploit the characteristic pathological features of the tumour microenvironment, and as such are prone to debilitating systemic toxicities. This review examines strategies for tumour microenvironment targeting to improve nanoparticle delivery, with particular focus on the delivery of nucleic acids and gold nanoparticles. Evidence for key research areas and future technologies are presented and critically evaluated. Among the most promising technologies are the development of next-generation cell penetrating peptides and the incorporation of micro-environment responsive stealth molecules.

## Nanoparticles in the clinic

In the last 20 years, nanoparticle delivery systems have been at the forefront of research for improving cancer therapeutic delivery and efficacy. A number of nano-therapies have been FDA approved for the treatment of cancer such as Doxil® and Vyxeros®, with approved therapies most often being liposomal in nature, delivering pre-approved chemotherapeutics. However, despite the wealth of promising pre-clinical data for the use of nanoparticle therapeutics, successful clinical translation has proved challenging. One example of this is Aroplatin™ a platinum analogue (cis–trans-R,R-1,2- diaminocyclohexane decanoate platinum (II) encapsulated within a liposome. In vivo, Aroplatin was shown to successfully increase survival of mice bearing L1210 leukemia following both intravenous and intraperitoneal administration, compared to cisplatin delivered as a free drug. Aroplatin also increased the survival time of mice bearing reticulosarcoma liver metastasis by 37% over free drug (Perez-Soler et al. 1987). However, in the clinical setting benefits of Aroplatin were modest with only 5.6% of patients displaying a partial response (Liu et al. 2013). Additionally, concerns with regards to nanoparticle stability and potential toxicity arose due to large lipid loading and critical organ accumulation. More recently, Livatag® doxorubicin nanoparticles used in the treatment of hepatocellular carcinoma exhibited superiority in pre-clinical studies but failed to deliver patient benefit in phase III studies over standard therapeutics such sorafenib, gemcitabine and oxaliplatin. As such Livatag® clinical trials were halted.

A number of common factors inhibit the transition of nanoparticle therapeutics from the bench to the bedside. These include formulation complexity, pharmaceutical stability (biocompatibility and degradability), poor methodology of pre-clinical studies (e.g. randomisation), upscaling and a lack of standardisation with regards pre-clinical validation (Hua et al. 2018). Another major contributing factor is an over-reliance on passive targeting, exploiting the widely cited enhanced permeability retention effect (EPR). Typically this approach results in < 1% of the systemic administered dose reaching the target destination (Wilhelm et al. 2016). Importantly, insufficient tumour specificity leads to greater systemic toxicity, poor disease control and premature termination of clinical trials. An example of this occurred using an albumin-bound paclitaxel nanoparticle for the treatment of refractory multiple myeloma. Withdrawn in November 2014 (NCT01646762), the trial studied the therapeutic efficacy of nab-paclitaxel nanoparticles on 13 patients who had all previously failed with several rounds of different chemotherapies. Of the 13 patients enrolled 2 patients presented with partial disease response, with 6 patients displaying signs of disease progression. Given the advanced state of disease progression of these patients a reasonable 3.7 month increase in survival was shown. However, the trial was severely limited by a series of adverse side effects, ultimately leading to termination of the study. This was demonstrated in the fact that 85% of patients experienced peripheral sensory neuropathy, with > 50% of patients developing severe nausea. In one instance a patient developed grade 5 sepsis, influenced by drug-induced neutropenia which ultimately proved fatal. However, since the withdrawal of this trial, the use of nab-paclitaxel has been explored and NICE approved for the treatment of pancreatic cancer, where long-term prognosis is particularly poor. These and other similar example serve to highlight the importance of incorporating tumour-responsive elements into nanoparticle design to help limit systemic toxicities while simultaneously enhancing therapeutic efficacy.

## The tumour microenvironment

Cancers arise from genetic mutations, typically characterised by uncontrollable cell proliferation that results in the formation of a neoplastic mass, that can lead to secondary metastatic lesions (Hanahan and Weinberg 2011). As such, most early cytotoxic chemotherapeutic agents were designed to preferentially destroy cells undergoing continuous proliferation. However, standard chemotherapeutics such as docetaxel, doxorubicin and gemcitabine often result in minimal therapeutic differentials, causing widespread toxicity, with modest benefits in terms of overall survival (Pan et al. 2014; Huanwen et al. 2009). Therefore, it is contingent upon scientists to exploit the ever-increasing knowledge of the unique tumour microenvironment (TME), to designing the next generation of targeted therapeutics.

**Fig. 1:**
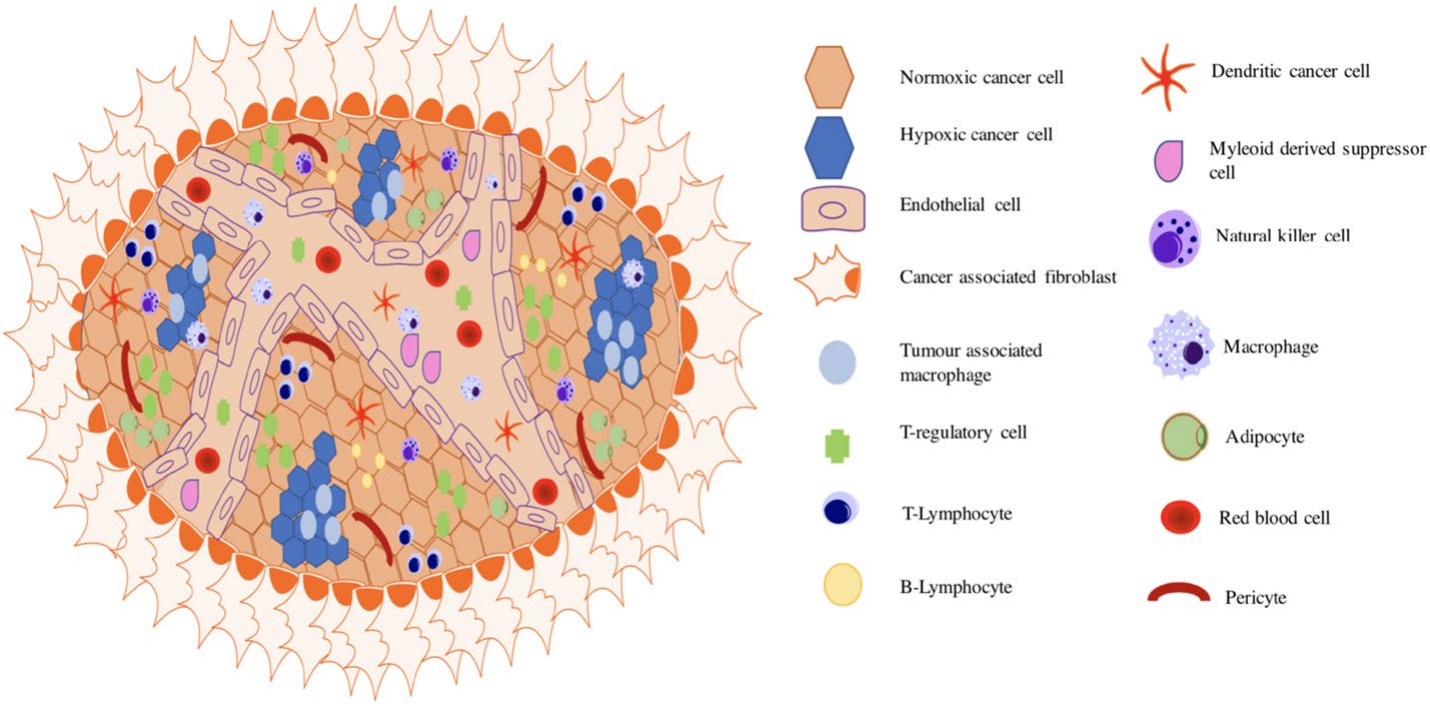
Schematic representation of the cells and vasculature of the tumour microenvironment. There are several targets within the TME that distinguish tumour tissue from healthy tissue. These include: cell profile, vasculature, altered oxygenation status and

The TME is a dynamic network of immune, stromal, tumour and endothelial cells, the latter of which form a dysfunctional vasculature producing elevated secretion of inflammatory and growth signalling molecules (Fig. 1, Table 1). The complex signalling interplay within the TME contributes to neoplastic survival and growth by supplying nutrients, O_2_ and stimulatory factors. In contrast to healthy tissue, the TME is characterised by a high interstitial fluid pressure (IFP) and a low intracellular pH (pH between 6.0 and 7.0), both of which impede the delivery and efficacy of current therapeutics (Kato et al. 2013). Additionally, the complexity of the tumour microenvironment (TME) has been shown to result in premature degradation of cargo, reduction in circulation times and poor target cell uptake. Despite this, the physiological differences of the TME can be exploited using nanotechnology to ensure targeted delivery of therapeutic cargoes. Within this review, the latest developments in nano-therapeutics are discussed along with potential pitfalls and areas for future development.

**Table 1:** Cellular involvement in the tumour microenvironment

Cell type	Role	References
T-lymphocytes	CD8^+^: cytotoxic, good for prognosis CD4^+^: Th1—production of IL-2 and IFN-γ. Important for immune defense CD4^+^: Th2—tumour promoting, linked with inflammatory phenotype. Secrete inflammatory cytokines. Poor prognosis if in high number	Botchway et al. (2015), Brown et al. (2010), Burroughs et al. (2013)
T-Regulatory (T-Reg) (lymphocyte)	Tumour promoting/ suppress tumour immunity Produce IL-10 and TGF-β allowing for enhanced cell growth Reduce cellular response to oxidative stress thereby contributing to the development of therapeutic resistance	Cathcart et al. (2015), Cheng et al. (2017), Coulter et al. (2013)
Pericytes	Contractile cells Differentiate to stromal fibroblasts contributing to invasion/metastasis Provide structural support for blood vessels Decreased expression in TME allowing for increased metastasis	Cox et al. (2014), Dai et al. (2011)
B-lymphocytes	Located in the invasive margin of the tumour and lymph nodes Involved in antitumour humoral immunity Release of cytokines and lymphotoxin activating pro-inflammatory pathways such as NF-κB	Deshayes et al. (2005), Dixit et al. (2015), Erler et al. (2006)
Natural Killer (NK)	Innate cytotoxic lymphocytes Normally powerful cytotoxic activity but decreased presence in TME Influences a tumours ability to control tumour growth Important role in response to some targeted antibody therapies such as trastuzumab	Fais et al. (2014), Feuerecker et al. (2015)
Adipocytes	Aid recruitment of malignant cells due to presence of free fatty acids Act as “fuel” for cancer cells Assist in recruitment of macrophages, polarizing to M2 phenotype Produce IL-6, CCL2 and TNF-α	Gao et al. (2017), Gialeli et al. (2011), Haley and Frenkel (2008)
Dendritic	Normal immune function—antigen presenting and processing cells Reduction in antigen presenting function Accumulation within tumour associated with increased patient survival	Hamdan and Zihlif (2014), Hanahan and Weinberg (2011)
Tumour associated neutrophils (TAN)	Promote primary tumour growth Enhance angiogenesis Facilitate ECM degradation ROS and RNS production—DNA damage	Hatakeyama et al. (2007), Heitz et al. (2009), Heldin et al. (2004), Hill et al. (2008)
Tumour associated macrophages (TAM)	Highly expressed in hypoxic, necrotic areas Associated with poor prognosis Involved in cell migration, invasion and metastasis and epithelial-mesenchymal transition Increase expression of MMP	Hua et al. (2018), Huanwen et al. (2009), Kanapathipillai et al. (2012)
Myeloid derived suppressor cell(MDSC )	Inhibitory immune cell Promote tumour growth Inhibit CD8^+^ T cell activity by increasing NOS2 expression	Hamdan and Zihlif (2014), Kato et al. (2013), Kobayashi et al. (2014)
Vascular endothelial	Line the lumen of blood vessels essential for nutrient/oxygen supply In TME—abnormal in shape, chaotic branching promoting a leaky vasculature Stimulate inflammation and metastasis	Kumar et al. (2013), Li et al. (2013)
Cancer associated fibroblasts (CAF)	Involved in organ fibrosis and cancer development Secrete chemo-attractants and growth factors—e.g. CXCL12 promotes growth and survival of malignant cells Enhances MMP production and neovascularization	Li et al. (2004, 2016), Liu et al. (2013)

## TME and vasculature

### Nanoparticle therapy and high IFP

Metallic and non-metallic nanoparticles hold significant potential for use in diagnostic imaging, as radiosensitisers and as a means of repurposing existing therapeutics (Botchway et al. 2015; Brown et al. 2010). Nanoparticles preferentially accumulate in tumour tissue over healthy tissue due to the enhanced permeability and retention (EPR) effect (Kobayashi et al. 2014; Nakamura et al. 2016; Ngoune et al. 2016). Growing tumours require vasculature, however, the tumour vascular network fails to undergo sufficient vascular remodelling, as a result dysfunctional vasculature with poor lymphatic drainage forms. Impaired lymphatic drainage results in an imbalance of molecular fluid pressure creating high interstitial fluid pressure (IFP) ranging from 40 to 60 mmHg, compared to between 3 and 10 mmHg in healthy tissue (Omidi and Barar 2014).

Interstitial fluid pressure regulates transcapillary flow, therefore, influencing the uptake of high molecular weight compounds such as chemotherapeutics that are transported via convection (Heldin et al. 2004). Salnikov et al. (2003) reported that high IFP impedes transcapillary transport, uptake and therapeutic efficacy of traditional cytotoxic chemotherapeutics such as 5-Fluorouracil (5-FU). Using a Wistar-Moller rat mammary carcinoma model, the authors demonstrated that reversal of high IFP using *s.c.* injection of Prostaglandin E1 (PGE_1_) resulted in a 40% increase in 5-FU uptake compared to rats receiving no pre-treatment. Despite this the effect was transient, remaining for a maximum of 1 h post PGE_1_ administration. This suggests that repeated dosing of PGE_1_ would be necessary to maintain enhanced 5-FU uptake and efficacy, highlighting practical limitations to this approach (Salnikov et al. 2003).

An alternative to this strategy is to develop hybrid nanoparticle drug conjugates. Nanoparticles are attractive due to their versatility as can be modified in terms of both size, surface charge and functionalisation to take advantage of the TME and high IFP enabling greater target site drug accumulation.

Gao et al. (2017) examined how nanoparticles can be used not only to enhance chemotherapy delivery through the EPR effect but by also reducing tumour IFP. In this instance the authors synthesised a complex tumour-responsive gelatin-coated lipid nanoparticle (GNP) designed to reduce tumour IFP while simultaneously enhancing the tumour specific release of the chemotherapeutics docetaxel and quercetin. These two chemotherapeutics were encapsulated within a lipid core composed of glycerine monosterate, egg phosphatidyl choline and capric triglyceride. The lipid core is encapsulated with an outer gelatin layer which contains the tyrosine kinase inhibitor imantinib. Imantinib was used to reduce tumour IFP by inhibiting the expression if Bcr-Abl and platelet derived growth factor (PDGF), blocking interactions between the extracellular matrix and cancer-associated fibroblasts, thus reducing IFP. The outer particle layer was composed of gelatin, enabling tumour responsiveness, the gelatin layer is degraded by matrix metalloproteinases (MMP), overexpressed within the TME. Importantly, this approach improves drug-specific release within the TME, avoiding off-target toxicity. MTT viability assays demonstrated that the nanoparticle system significantly reduced the IC_50_ concentration for the combination of docetaxel and quercetin (6.18 ± 0.35 μg/ml) compared to that of free drug alone (13.43 ± 0.8 μg/ml). The authors attribute this increased efficacy to enhanced target cell internalisation, however, uptake studies show less endocytosis of the gelatin-coated nanoparticle compared to the lipid only particle. It is, therefore, likely that the increased toxicity is due to the inclusion of imantinib. Imantinib reduced tumoural IFP by > 40% (17.2 mmHg) in a nude 4T1 xenograft mouse model, with control animals exhibiting an IFP of 29.18 mmHg. Importantly, the impact of both in vitro enhanced toxicity and in vivo reduced IFP translated to a significant anti-tumour effect with a 2.8 fold increase in tumour growth delay and a 72.5% reduction in the formation of pulmonary metastases for GPN treated animals (Docetaxel, Imantinb 10 mg kg, quercetin 5 mg kg) (Gao et al. 2017). This study outlines a potentially promising tumour-specific delivery vehicle for chemotherapeutics that have previously been shown to induce off-target toxicity. The inclusion of gelatin and imantinib theoretically enable tumour specificity, however, limited information with respect to in vivo biodistribution, particularly within the liver and spleen, leave unanswered questions with respect to targeting efficacy, and the full clinical potential.

### Tumour vasculature and hypoxia

Accelerated proliferation coupled with a deficient vasculature often results in dynamic tumour hypoxia. Tumours adapt to chronic hypoxia inducing changes in gene expression through the activation of pro-survival and metastatic genes such as nuclear factor kappa beta (*NF*-*κB*), Hypoxia-inducible factor-1-alpha (*HIF*-*1*-*α*), *PIM*-*1* oncogene (*PIM*-*1*) and endothelin-1 (*EDN 1*) (Hamdan and Zihlif 2014; Dai et al. 2011). In turn, hypoxia has been directly correlated to an aggressive, metastatic tumour phenotype with hypoxic tumours generally exhibiting resistance to conventional cancer treatments (Rockwell et al. 2009). Tumour hypoxia has been harnessed in several different ways for the treatment of cancer, including the development of nano-formulations to enhance the delivery of encapsulated chemotherapies. Thambi et al. (2014) assessed the use of self-assembled hypoxia smart nitroimidazole-dextran nanoparticles to enhance the delivery and therapeutic effect of the chemotherapeutic doxorubicin (DPX-HR-NP). Under low oxygen tensions, the nitro group of nitroimidazole is converted to an amino group, altering the hydrophilicity of the nanoparticle complex. The authors report that this conversion allows for a water-soluble particle, enabling an enhanced release of hydrophobic drugs in hypoxic conditions. The authors demonstrated a 40% increase in doxorubicin release under hypoxic conditions compared to normoxia when encapsulated in the nitroimidazole-dextran nanoparticle. This corresponded to an increased tumour-specific in vivo accumulation of DPX-HR-NP, four-fold greater than measured in any normal organ. Unsurprisingly, DPX-HR-NP also significantly reduced tumour volume of SCC7 bearing nude mice when delivered intravenously, compared to that of mice treated with an equal concentration (5 mg/kg) of free doxorubicin, observing time-matched tumour volumes of 700 mm^3^ and 300 mm^3^, respectively (Thambi et al. 2014).

The hypoxic properties of the TME can be directly targeted using siRNA therapeutics. Hypoxia-inducible factor-1-alpha (HIF-1-α) is overexpressed under hypoxic conditions, driving a corresponding increase in the expression of downstream targets such as vascular endothelial growth factor (VEGF), involved in angiogenesis, glucose metabolism and invasion (Burroughs et al. 2013). Successful inhibition of HIF-1 has been demonstrated in both prostate and lung cancer using chemical inhibitors such as PX-478 and topotecan. However, clinical effectiveness is greatly reduced by serious adverse effects including neutropenia and acute weight loss, as such alternative strategies have been pursued (Welsh et al. 2004). Liu et al. (2012) investigated the use of siRNA targeted to HIF-1-α to suppress expression and inhibit the growth of hypoxic prostate tumours (Liu et al. 2012). HIF-1-α siRNA was delivered through self-assembled micelle nanoparticles composed of two polymers, poly-ε-caprolactone and poly-2-aminoethylethyenephosphate. Nanoparticles were 58 ± 3.4 nm in diameter with a surface charge of 24.3 ± 2.31 mV. Maximal inhibition of HIF-1 expression was demonstrated using 400 nM of HIF-1-α siRNA, resulting in reduced tubule formation and enhanced wound closure compared to untreated, hypoxic PC-3 cells. Furthermore, suppression of HIF-1 inhibited PC-3 xenograft growth in athymic mice following *i.v*. injection with 2 mg of siRNA loaded nanoparticles every second day over 22 days. At the experimental endpoint, siRNA treated tumour volume was measured as 280 mm^3^ compared to 500 mm^3^ for mice treated with blank nanoparticles (Liu et al. 2012). This study highlights the potential therapeutic benefit of HIF-1 inhibition in hypoxic tumours. However, HIF-1 inhibition may not be a viable therapeutic approach for all patient cohorts. Zhang et al. (2010) have shown that inhibition of HIF-1 negatively impacts the normal angiogenic response with HIF-1 heterozygous null mice exhibiting delayed wound healing, a side effect that would be particularly problematic in elderly or diabetic patient cohorts (Zhang et al. 2010). However, as the effects of siRNA therapeutics are short-lived and transient, hypoxia-induced treatment resistance could be overcome, enabling the enhanced efficacy of traditional therapies, while limiting the impact on the normal angiogenic response.

## Acidosis

The TME is characterised as possessing a lower pH compared to physiological tissue, a result of excess lactic acid from altered tumour cell metabolism. The pH of the TME can be as low as 5.5, driven by a phenomenon commonly known as the Warburg effect (Fais et al. 2014). The acidic TME has been shown to drive tumour progression and development (Feuerecker et al. 2015). Rofstad et al. (2006) highlighted that tumour cell acidosis significantly increased the formation of pulmonary melanoma metastases in nude athymic mice, demonstrating that increased metastasis occurs as a consequence of the upregulation of proteolytic enzymes such as MMP-2/9 and the pro-angiogenic protein VEGF-A. Proteinase enzymes such as MMP-2/9 have been shown to have optimal activity at low pH, with activity being upregulated as a consequence of increased phospholipase D, MAPK and NFκB signalling (Kato et al. 2013). Antibody inhibition of VEGF-A decreased angiogenic potential by 2.1–3.8-fold, with invasion through Matrigel^®^ chamber assays being reduced when a known MMP-2/9 inhibitor was used. MMP2/9 inhibition was also shown to reduce the formation of pulmonary metastases in vivo (Rofstad et al. 2006).

Disruption of the acid/base balance perturbs the uptake of chemotherapeutics, which are often acidic or basic in nature, contributing to treatment failure and resistance. For example, Swietach et al. (2012) highlighted a twofold decrease in doxorubicin uptake in HCT116—colorectal cells—when cultured at pH 6.4 compared to pH 7.4. The authors attribute this reduction to pH partitioning, contributing to the ionisation of doxorubicin and ion trapping, thereby reducing membrane permeability (Swietach et al. 2012). Proton pump inhibitors such as omeprazole have been used to reduce tumour cell acidosis, but studies have shown little clinical benefit (Marino et al. 2010). Consequently, efforts are now focused on exploiting the acidic tumour microenvironment to improve the delivery of nanoparticle-encapsulated chemotherapeutics.

Li et al. (2016) covalently attached a cisplatin pro-drug to a poly(amidoamine) (PAMAM) dendrimer block containing tertiary amine groups. Nanoparticles were formed via dialysis, removing any unbound polymer, with a mean size of 80 nm at neutral pH. However, under slightly acidic conditions the tertiary amines become protonated allowing for rapid platinum disassociation from the dendrimer block, evidenced by a reduction in NP size to 10 nm. The authors hypothesised this pH-sensitive system would allow for increased tumour-specific cisplatin uptake, enhancing its efficacy while limiting systemic toxicity.

Supporting evidence for this hypothesis was demonstrated with increased platinum accumulation in Bx-PC3 (pancreatic cancer) spheroids, with up to a sevenfold increase in intratumoural platinum compared to free cisplatin or cisplatin delivered using a non-pH sensitive system. Furthermore, use of this vehicle translated to significant tumour growth delay in a Bx-PC3 xenografts by 82%, compared to 24% for mice treated with free cisplatin at a 2 mg/kg dose (Li et al. 2016). Yet, the clinical potential of this delivery vehicle for the treatment of pancreatic cancer can be debated. Throughout this study, only one pancreatic adenocarcinoma cell line was utilised—Bx-PC3. This cell line cannot be considered as a universal model for pancreatic cancer due to the absence of a K-ras mutation. Indeed, approximately 90% of the pancreatic patient cohort possess mutations in the K-ras oncogene (Cox et al. 2014). Therefore, to assess the translational efficacy of the delivery vehicle in pancreatic cancer further studies in ras mutated cell lines such as panc-1 and Mia-Paca-2 would be essential. Furthermore, tissue acidosis does not only occur in tumour tissue, anaerobic glycolysis creating a build-up of lactic acid can occur in numerous parts of the body including muscle tissue. Therefore, if the nanoparticle lacks tumour specificity it may accumulate within unwanted tissues leading to unwanted side effects.

One approach to circumvent this problem is to conjugate a tumour specific targeting ligand to the nanoparticle, an example of which was demonstrated by Cheng et al. (2017). The authors developed a pH-responsive folic acid conjugated polydopamine modified mesoporous silica nanoparticle for the targeted delivery of doxorubicin. This formulation enables tumour specificity due to the presence of the folic acid group. Folic acids have high affinity for folate receptors which are overexpressed on the surface of cancer cells. Particle size was demonstrated to be 193.08 ± 8.1 nm with a zeta potential of − 4.8 ± 0.9 mV. Drug release studies highlighted that 49.5% of the total doxorubicin loaded was released at a pH of 2 over a 190 h period, compared to only 28.5% at pH 7.4, highlighting the acid sensitivity. In vivo biodistribution studies demonstrated greatest accumulation of the MSN-Dox-PDA-PEG-FA in the tumour compared to other organs, 24 h post systemic injection. However, accumulation was still evident within the kidney and liver, particularly at the earlier time points suggesting that folic acid targeting may not be specific enough to prevent accumulation in normal organs. Despite this, increased doxorubicin efficacy using the mesoporous nanoparticle was observed following systemic administration. At experimental endpoint (16 days) Hela tumour volume in female athymic mice treated with the MSN-Dox-PDA-PEG-FA nanoparticle was reported to be fourfold lower than mice treated with 5 mg/kg of free doxorubicin (~ 400 mm^3^) and tenfold lower than mice treated with saline alone(~ 1000 mm^3^) (Cheng et al. 2017).

This delivery system is a good example of how TME acidosis and active targeting can achieve enhanced chemotherapeutic uptake. Hydrodynamic particle size and zeta potential are important characteristics that influence the internalisation and therapeutic efficacy of nanoparticle-based therapeutic. A positive surface charge is reported to be important for increasing nanoparticle endocytosis. This is because a positive surface charge facilitates target cell contact due to electrostatic interaction towards negatively charged proteoglycans located within the plasma membrane. However, a balance must be met as strongly cationic particles have been shown to induce unwanted toxicity (Verma and Stellacci 2010). Particles less than 200 nm in diameter are optimally sized for uptake via clathrin-mediated endocytosis. Chithrani et al. (2010) reported that AuNP size is an important determinant factor for uptake, demonstrating that 50 nm transferrin coated AuNP were more readily internalized than 10 and 100 nm particles due to the “wrapping effect” (Zhang et al. 2014). The wrapping effect is the process by which the cell membrane encapsulates nanoparticles, independent of receptor/ligand interactions (Bishop et al. 2009). Additionally, developing sub-200 nm functionalised particles is important as endocytosis above this size is dominated by caveolae-mediated endocytosis (CME) (Verma and Stellacci 2010). The relevance of this is that many tumour sub-types and cell lines including PC-3 (prostate), HT29 (colon carcinoma) and SK-BR3 (breast) exhibit decreased expression of caveolin-1, an essential protein required for the formation of the plasma membrane and caveolin vesicles (Hill et al. 2008; Bender et al. 2000). Therefore, ensuring that particle size remains sub-200 nm should circumvent the reduced endocytic potential associated with this tumour-specific alteration. Another strategy to improve the physical characteristics of a delivery system and to further improve drug uptake is to conjugate the nanoparticle to a cell penetrating peptides, discussed further in “Therapeutics targeting the TME”.

## Immune component

Tumours are inherently immunogenic exhibiting a significant immune cell infiltrate (Fig. 1 and Table 1). In cancers such as glioblastoma, inflammation and an upregulated immune response are strongly correlated with enhanced invasion and metastasis (Baek et al. 2011). However, successful nanoparticle delivery in the cases of brain tumours face the additional barrier of overcoming the blood–brain tumour barrier (BBTB). Recent studies have demonstrated that it may be possible to exploit the immune cell profile of the TME, piggybacking leukocyte infiltration to enhance NP uptake across the BBTB.

Pang et al. (2016) observed that macrophage recruitment is markedly enhanced in glioblastoma tumours, with up to a third of the tumour mass composed of macrophage cells. The authors hypothesise that using macrophages as a delivery vehicle for doxorubicin-containing nanoparticles (M-DOXNP) will result in increased tumour penetration and therapeutic effect compared to doxorubicin delivered in nanoparticle form alone. To demonstrate the ability of macrophages to deliver DOXNP, the authors pre-treated Raw264.7 murine macrophage cells with DOXNP. Nanoparticles ranging between 100 and 200 nm were most avidly phagocytosed causing no direct toxicity. Using a U87 glioma spheroid model the authors demonstrated increased penetration of the M-DOXNP, 56.4 μm from outer rim of spheroid in comparison to the DOXNP which only penetrated 36.07 μm. Importantly, in vivo increased circulation times and accumulation within the glioma parenchyma were demonstrated using M-DOXNP compared to DOXNP alone, highlighting the potential of exploiting the immune component of the TME for NP delivery (Pang et al. 2016). Despite this, several challenges remain with this type of delivery system. The authors demonstrated that only 42% of doxorubicin loaded into the nanoparticles was released over a 24 h period using the macrophage delivery. As such, drug loading would need to be increased to achieve a full therapeutic effect, which may directly trigger toxicity within the macrophage cells. Additionally, the clinical translation of the approach would prove challenging in that patient cells would require pre-harvesting prior to nanoparticle loading and re-administration. Although promising, this approach remains very much at early stage pre-clinical development.

## Extracellular matrix

The extracellular matrix (ECM) is composed of the basement membrane and interstitial matrix, providing tissue structure, regulating cell proliferation and migration (Lu et al. 2012). ECM turnover is tightly regulated in healthy tissue. However, ECM dysregulation is commonly observed in tumours due to aberrant enzyme expression, leading to increased stiffness of the ECM, as well as degradation of the basement membrane (Gialeli et al. 2011). To date, relatively little is known about the therapeutic potential of targeting the ECM in the treatment of cancer. However, as the ECM is not surrounded by a plasma membrane nanoparticle therapeutics are an attractive delivery vehicle with no extracellular barrier to uptake.

Lysyl oxidase (LOX) is an enzyme responsible for the cross-linking of collagen to elastin in the ECM, inducing ECM rigidity. LOX has been shown to be elevated in numerous tumour subtypes including breast and head and neck cancer (Erler et al. 2006). Overexpression of LOX contributes to the development of a metastatic niche through the induction of proliferative signalling pathways such as NF-κB (Mayorca-Guiliani and Erler 2013). Kanapathipillai et al. (2012) suggests that inhibition of LOX confers therapeutic benefit in the treatment of mammary gland cancer, with the most promising approach to date being anti-lox monoclonal antibodies. However, the clinical potential of anti-LOX antibodies has largely been impeded by the high concentrations of antibody required to induce a therapeutic effect, resulting in a series of unwanted side effects. Subsequently, LOX_Ab_ were conjugated to an amphiphilic poly (lactide-co-glycolide-block-poly (ethylene glycol) (PLGA-b-PEG-COOH) nanoparticle. Conjugation via covalent carbodiimide chemistry produced 220 nm nanoparticles determined by dynamic light scattering (DLS) and TEM. At a nanoparticle/LOX_Ab_ concentration of 50 μg/ml, a fourfold inhibition of 4T1 cell proliferation and migration was demonstrated. Additionally, the proliferative rate was unaffected when using an equal concentration of free soluble antibody. Importantly, the nanoparticle conjugated antibody resulted in a 25-fold reduction in dose to suppress an orthotropic mammary 4TI mouse tumour model compared to free antibody. Conjugation also resulted in an increased therapeutic index, with a decrease in adverse effects (Kanapathipillai et al. 2012). However, in vivo biodistribution studies demonstrated limited tumour specificity, with fivefold higher liver accumulation over tumour following systemic injection. Modifications to this delivery system are, therefore, required for increased tumour specificity. Potential modifications may include incorporation of a tumour targeting motif or optimisation of the PEG size.

Another key feature of the tumour ECM is evidence of degradation. This degradation occurs as a consequence of elevated levels of matrix metalloproteinases such as MMP-2 and MMP-9. ECM degradation has been shown to enhance the proliferation and metastasis of cancer cells, with MMP expression correlated to advanced, treatment refractory phenotypes, in a number of cancers. However, unlike the LOX enzyme there is little evidence of nanoparticle therapies designed to reduce its expression. Instead, a wealth of literature is presented as to how its expression can be exploited to enhance nanoparticle delivery, stability and drug deposition. Cleavable MMP sequences have been developed as a means of removing stealth molecules when a nanoparticle reaches the TME. MMP cleavable sequences have been attached to mesoporous silica nanoparticles (MSN) to enhance the uptake and therapeutic efficacy of cytotoxic chemotherapeutics. For example, Rijt et al. (2015) conjugated an MMP-9 responsive linker to avidin capped MSNs to enhance the uptake and efficacy of cisplatin in a 3D- lung cancer tissue model. The authors demonstrated that cisplatin release from the MSN only occurred when recombinant MMP-9 was added, leading to cleavage of the avidin cap.

Tissue extracted from cisplatin-treated k-ras mutated transgenic mice showed increased cell death of up to 20-fold, with a corresponding increase in apoptosis (Van Rijt et al. 2015). Furthermore, data from the same study showed that when nanoparticles are placed on top of the tissue, cisplatin is released and is able to diffuse through the sample inducing its therapeutic effect. This may be beneficial as it would remove the requirement for additional targeting molecules to ensure nanoparticle internalisation. However, this approach may be less effective in large solid tumours with hypoxic cores, where transcapillary transport is reduced limiting drug diffusion.

## Therapeutics targeting the TME

### Gene therapy

Cancer can be described as a disease of genetic mutations, aberrant signalling and DNA damage. Consequently, gene therapy has been proposed as an effective treatment regime, with 64% of current gene therapy clinical trials designed for the treatment of cancer. However, the clinical potential of gene therapy is greatly reduced by the barriers faced when delivering oligonucleotide therapeutics. Nucleic acids are vulnerable to rapid degradation by the reticuloendothelial system (RES). RES, a component of the innate immune system, recognises and clears foreign pathogens and particles through opsonisation and phagocytosis. Additionally, DNA is both hydrophilic and anionic in nature, and therefore, is repelled from the phospholipid bilayer of cell membranes, impeding the movement of the DNA into the cytosol (Loughran et al. 2015). Once DNA is internalised endosomal entrapment can arise with DNA becoming lysed and degraded prior to release, rendering its therapeutic potential void (McErlean et al. 2016). Therefore, it is essential that gene therapy constructs must be actively targeted to the tumour.

Targeting the TME using nanoparticles as delivery vehicles for nucleic acids has been explored as a means of increasing tumour accumulation and efficacy. Nucleic acid nanoparticles have been actively targeted to tumour cells by targeting elevated expression of folate and transferrin receptors found on the surface of many tumour cells (Zwicke et al. 2012; Dixit et al. 2015).

It is well known that the loss of the tumour suppressor p53 is responsible for tumour survival and the development of treatment refractory phenotypes in a wide variety of cancers (Sigal and Rotter 2000). Consequently, reintroduction of functional p53 could prove beneficial for patient survival and treatment outcomes. Senzer et al. (2013) incorporated a plasmid encoding for wild-type p53 (wt-p53) in a *N*-[1-(2,3Dioleoxloxypropyl]-*N*–*N*,Ntrimethylammonium (DOTAP): 1,2Dioleoyl-sn-glycero-3-phosphoethanolamine (DOPE) liposomal nanoparticle, conjugated to an anti-transferrin antibody, enabling tumour specific targeting. Patients with a variety of solid tumours including colorectal and pancreatic received treatment with the nanoparticle conjugates on a dose escalating regime from 0.6 to 3.6 mg of DNA per infusion. Post treatment, PCR analysis highlighted tumour-specific increased expression of p53, with negligible increases observed in normal skin biopsies, suggesting that transferrin confers tumour specificity. 7 out of 11 patients within the study displayed stable disease at a 6-week post-treatment, with an increased median survival of 340 days. However, only one patient displayed evidence of tumour necrosis, demonstrating the limited effect of SGT-53 as a monotherapy (Senzer et al. 2013). Subsequently, phase one clinical trials have been completed assessing the effectiveness of SGT-53 in combination with docetaxel, in patients who previously failed to respond to taxane chemotherapy. This study enrolled 14 patients with metastatic refractory disease administering 10 infusions of SGT-53 (2.4/3.6 mg) and 3 infusions of docetaxel (40, 60 or 70 mg/m^2^). Preliminary results indicated that 2 patients experienced a partial response, with 67% of patients displaying stable disease (Pirollo et al. 2016). Further phase II clinical trials determining the effectiveness of SGT-53 in combination with Gemcitabine/Nab-Paclitaxel (NCT02340117) in the treatment of metastatic pancreatic cancer are now underway, with results expected in early 2019.

Using targeting ligands, and in particular transferrin specific targeting, has helped to overcome intracellular barriers faced when delivering nucleic acid and gene therapy (Niidome and Huang 2002). However, premature degradation of targeting molecules can occur upon systemic delivery resulting in non-specific protein binding and the formation of protein corona, triggering phagocytosis and excretion.

#### Cell-penetrating peptides

Cell-penetrating peptides (CPP) are short peptide sequences that facilitate cell internalisation through a variety of endocytic pathways, without the requirement of specific targeting receptors (Heitz et al. 2009). CPP are known to facilitate the delivery of numerous nanoparticle cargoes in a variety of cell lines and in vivo models, with peptides being easily modified depending on the cargo being delivered (Deshayes et al. 2005).

Early naturally derived CPP such as TAT (sequence: GRKKRRQRRR) and Penetratin (sequence: RQIKIYFQNRRMKWKK) are composed of basic amino acids enabling an alpha-helical conformation and translocation of cargo across the cell membrane. Extensive research has been carried out into the key components of a CPP structure. Mitchell et al. (2000) highlighted the importance of the number of arginine residues within a peptide with respect to penetrating potential. Using fluorescin labelled oligoarginines, the authors observed poor endocytosis when incorporating less than 6 arginine residues, however, fluorescence increased exponentially when the peptide contained between 7 and 15 arginine residues. Increasing the arginine composition above 15 residues induced significant toxicity and conferred no additional uptake (Mitchell et al. 2000). However, the most promising CPP not only contain several arginine residues, but are also amphipathic in nature enabling both membrane interaction and pH responsiveness.

**Fig. 2:**
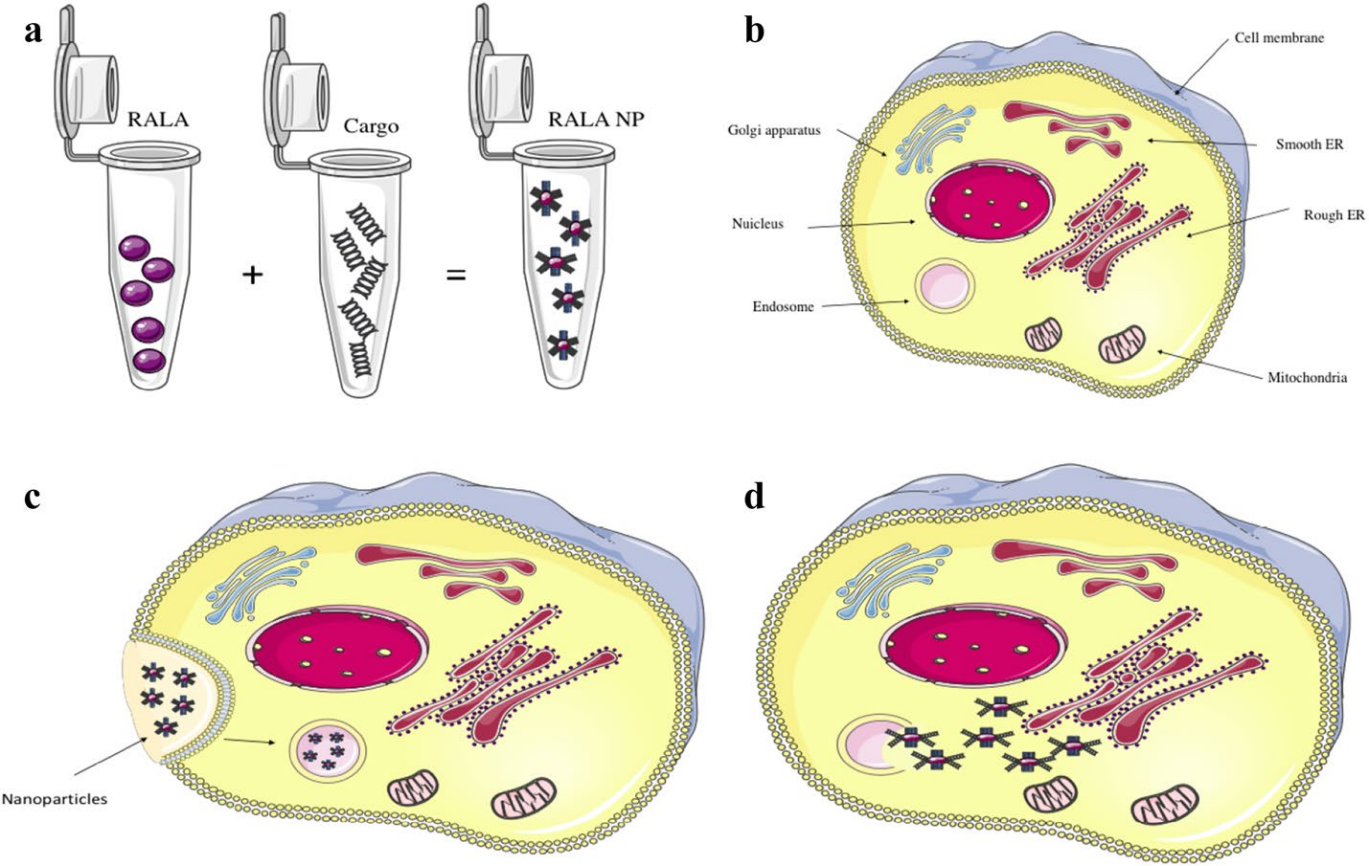
Cell penetrating peptide RALA enhances the delivery of therapeutic cargo. **a** RALA is complexed with negatively charged cargo, with particles forming through the formation of electrostatic interactions. **b** simple schematic of a cell. **c** Upon endocytosis nanoparticles can become trapped within the endosome. **d** RALA adopts an alpha-helical confirmation in the low pH of the endosome, allowing for interaction, disruption of the endosomal membrane and the release and trafficking of cargo to target tissues/organelles

One such peptide is RALA. RALA was derived from two alternative cell penetrating peptides termed GALA and KALA. GALA (sequence: WEAALAEALAEALAEHLAEALEALAA) is a fusogenic peptide with 7 identical glutamic acid–alanine–leucine–alanine repeats which provide the peptide with pH-responsive properties, enabling embedment within the phospholipid biolayer. However, the use of GALA as a delivery vehicle is limited by the fact that it cannot condense or bind to negatively charged moieties (Li et al. 2004). To address this, issue the glutamic acid was replaced with lysine giving rise to KALA, a positively charged peptide. KALA (sequence: WEAKLAKALAKALAKHLAKALAKALKACEA) which displays improved nucleic acid binding via electrostatic interactions, producing superior transfection efficiencies than observed with GALA. However, as KALA does not exhibit pH dependency of its alpha-helical conformation it was found to induce significant cytotoxicity as a consequence of cellular membrane interaction. McCarthy et al. (2014) developed RALA (sequence: WEARLARALARALALRHLARALALRARALRACEA), with the replacement of lysine to arginine, greatly improving cell penetration. RALA exhibits reduced toxicity due to its alpha-helical structure being pH-responsive, aiding cargo release through endosomal membrane interaction (Fig. 2). As such, RALA is a promising delivery platform for gene therapy, protecting cargo from unwanted degradation. McCarthy et al. (2014) have also shown that RALA produces transfection efficiencies equal to that of the market-leading transfection agent lipofectamine, but with the major advantage of less toxicity (Mccarthy et al. 2014; Bennett et al. 2015).

RALA has been shown to have the potential for the delivery of bisphosphonate drugs (BP’S), such as alendronate, which have bone-targeting specificity. This opens up the options of treating metastatic disease which form bone lesions. However, the clinical application of BP’s is impeded by systemic toxicity. Massey et al. (2016) present evidence that encapsulating BP’s into nanoparticle structures using RALA allows for improved delivery and the potentiation of anti-cancer effects. Using an MTS cell viability assay the authors highlight that BP’s which previously show no anticancer properties in the PC-3 cell line, induce significant cytotoxicity when delivered using RALA. Importantly, blank RALA nanoparticles induced no direct toxicity. RALA-alendronate particles, when directly injected into the tumour were demonstrated to delay tumour growth and increased survival by 56.3% in PC-3 xenografts (Massey et al. 2016). These studies highlight RALA, along with other CPP as an exciting delivery vehicle for a wide range of negatively charged moieties,

However, the biodistribution profile of RALA nanoparticles have highlighted high levels of accumulation in normal organs such as the lungs and liver, rather than the intended tumour (Mccarthy et al. 2014). This could potentially lead to the occurrence of off-target side effects. A similar distribution profile has been shown using alternative arginine-rich peptide delivery systems such as TAT and penetratin, suggesting that it is the high positive charge of the particles that lead to off-target accumulation (Nakase et al. 2012). However, particles possessing a neutral or negative surface charge display reduced cell-penetrating ability, therefore, a careful balance must be achieved. A potential strategy to reduce surface charge but still maintain a cationic property is through the inclusion of stealth molecules.

### Metallic nanoparticles

Metallic nanoparticles such as gold nanoparticles (AuNP) can take advantage of the leaky tumour vasculature, accumulating in tissue due to the EPR effect. This has led to a variety of possible applications including radiosensitisers, contrast agents and drug delivery vehicles (Coulter et al. 2013; McQuaid et al. 2016).

AuNPs have been used as delivery vehicles for cytotoxic chemotherapeutics such as 5-Fluorouracil, docetaxel and platinum-based chemotherapeutics such as oxaliplatin, all of which are hindered by low tumour-specific localisation, inefficient target cell internalisation and normal tissue toxicity. AuNPs are chemically inert and do not interfere with the structure or mechanism of a drug, but can enhance chemotherapy uptake and efficacy. Brown et al. (2010) conjugated the active region of oxaliplatin (Pt[R,R-dach]) to a PEGylated 31 nm AuNP. This resulted in particles of 176 nm ± 25 nm in diameter with a zeta potential of 14 mV ± 7.0 mV. In vitro, inductively coupled plasma mass spectrometry (ICP-MS) studies indicated that AuNP-oxaliplatin resulted in > twofold uptake enhancement in A549 lung carcinoma cells, compared to free oxaliplatin. The increased tumour cell internalisation of oxaliplatin, corresponded to an increase in efficacy with the LD_50_ concentration reduced by ~ 40% from 0.774 μM to 0.495 nM. (Brown et al. 2010).

Evidence within the literature also suggests that AuNP may have antiangiogenic activity and as such could be used to target specific proteins and receptors that are up-regulated within the TME. Mukherjee et al. (2005) hypothesised that due to the previous therapeutic use of gold in rheumatoid arthritis, that AuNP may prove useful inhibitors of angiogenesis. Using 5 nm AuNP the authors inhibited VEGF and basic fibroblast growth factor (bFGF) induced proliferation of both HUVEC and NIH3T3 cells, without triggering toxicity. At a dose of 670 nmol/l, the nanoparticles significantly decreased angiogenesis and oedema in a nude mouse ear experiment. Mechanistic studies demonstrated that this anti-angiogenic effect arose as a consequence of non-specific AuNP binding to the heparin domain of VEGF165 and BFGF, triggering a reduction in Rho signalling activity (Mukherjee et al. 2005). HUVEC (endothelial) and NIH3T3 (fibroblast) cells are abundant within the TME and play a crucial role in the signalling and growth response of tumours. Previous studies have shown AuNP to have minimal anti-proliferative activity and low toxicity, affirming their primary use as treatment sensitizers and drug carriers (Botchway et al. 2015).

Despite significant evidence presenting the potential clinical application of AuNP, a number of factors remain, impeding clinical translation. Metallic nanoparticles and particularly AuNP can be subject to rapid clearance through the reticuloendothelial system (RES) (Haley and Frenkel 2008). This is particularly true of unfunctionalised citrate-capped AuNP that are stable in neutral solutions such as phosphate buffered saline (PBS) due to electrostatic repulsion. However, when placed in full serum conditions (culture medium) or when systemically injected, non-specific protein absorption results in particle agglomeration, opsonisation and phagocytic clearance. In some cases, nanoparticles can accumulate in the liver and spleen, after which particle toxicity becomes a problem. The addition of stealth molecules on the surface of the particles has been shown to improve stability and consequently circulation and retention times.

### The PEG dilemma

Stealth polymers such as Poly-ethylene glycol (PEG) are hydrophilic in nature, coating hydrophobic nanoparticles and increasing particle stability through altered polarity (Liu et al. 2007). PEGylation prevents premature opsonisation and monocyte recognition thereby increasing circulation and retention time (Kumar et al. 2013). This is of particular importance in an in vivo context, enabling more predictable modelling of the biological response. However, studies have demonstrated that repeated administration of PEG can lead to a sensitised immune response and enhanced clearance rates. Yet, perhaps the biggest problem associated with the inclusion of stealth molecules is the negative impact on particle endocytosis, with increased polymer chain length conferring increased stability, but decreased uptake. Cruje and Chithrani (2015) assessed the uptake of 3 different AuNP preparations—(unPEGylated, 2 kDa PEG 50% coverage or 5 kDa PEG 50% coverage), in MCF-7 cells. Importantly, the incorporation of PEG attenuated particle uptake by up to tenfold in cells exposed to the 5 kDa version (Schellekens et al. 2013). This effect formed what is informally described as the “PEG dilemma”. It is, therefore, essential to refine the use of stabilising polymers to establish the maximum molecular weight/nanoparticle coverage that will enhance stability without impeding uptake.

Current efforts are focused on determining whether PEG molecules can be cleaved in a site-specific manner, enabling stability upon administration whilst also ensuring efficient endocytosis. One such approach involves PEG cleavage within the TME by exploiting tumour elevated levels of matrix metalloproteinase enzymes. Matrix metalloproteinases (MMPs) describe a class of extracellularly-expressed, zinc-dependent proteases that have a key role in physiological processes requiring ECM modification (Vartak and Gemeinhart 2007). In cancer, MMPs are responsible for the degradation of the basement membrane enabling release of growth factors such as VEGF, which promote proliferation and metastasis (Cathcart et al. 2015). Hatakeyama et al. 2007 exploited elevated levels of MMP-2 to promote cleavage of a 5K PEG on the MEND delivery system for nucleic acids (PPD-PEG-5K). Following *an i.v* injection in male nude BALB/c mice with a HT1080-Luc xenograft, a 70% reduction in luciferase activity was observed (Hatakeyama et al. 2007). Li et al. (2013) also developed an MMP responsive delivery system for gene therapy in the treatment of metastatic breast cancer (Li et al. 2013). The PAT-SPN nanoparticle was PEGylated to improve bioavailability, however, the PEG coating was cleaved once the nanoparticle reached a location rich in MMP-7. The authors demonstrated a 2.5-fold increase in target cell internalisation and therapeutic effect with the cleavable PEG nanoparticles compared to non-cleavable PEG nanoparticles and free siRNA alone. They attributed the increased cell uptake to exposure of the cationic corona which occurs upon PEG cleavage, enabling the siRNA to be internalised. Both studies suggest that exploiting environmental signals within the TME may prove beneficial for enhancing drug delivery with several other studies also suggesting this approach may prove beneficial for the delivery of metallic nanoparticles. However, the development and validation of a MMP responsive nanoparticle systems is challenging using in vitro systems. This is due to the fact that cells which exist in a homogeneous monolayer express low levels of enzymes such as MMPs compared to in vivo models. Consequently, it may be necessary to artificially induce expression, which in turn makes validation of enzyme responsive nanoparticle systems increasingly difficult.

An alternative approach to the cleavage of PEG within the TME is to exploit the acidic nature of the TME as previously discussed “TME and vasculature” section. Zhao et al. (2017) synthesised a “smart” cleavable pH sensitive PEG-s-PEI linker which was used to produce self-assembled nanoparticles encapsulating the chemotherapeutic docetaxel (DTX) and the non-steroidal anti-inflammatory drug indomethacin (IND). IND was used to limit weight loss often observed with cytotoxic therapies in both pre-clinical in vivo studies and in the clinic. The authors demonstrated PEG cleavage in acidic conditions (pH 7.4–6.5) through a decreased surface potential. PEG cleavage resulted in an increased release of DTX/IND over a 12 h period at pH 6.5 (max—0.4 nM/mg) compared to 0.2 nM/mg at pH 7.4 in the B16F10 tumour model. Furthermore, DTX/IND PEG-s-PEI nanoparticles delivered significant reductions in B16F10 and HepG2 tumour cell viability over free docetaxel at pH 6.5, confirming that cell acidosis contributes to treatment resistance and failure. Importantly, significant changes in tumour volume were demonstrated using a B16F10 xenograft when treated intravenously with the cleavable DOX/IND nanoparticle, compared to both free DTX and the non-cleavable DTX/IND-PEG-b-PEI nanoparticle. This decrease corresponded to the increased ability of the particle to exploit the TME, causing PEG cleavage that allowed increased docetaxel tumour specific accumulation (Zhao et al. 2017).

## Conclusions

As discussed throughout, exploiting characteristics of the TME for nanoparticle design and therapy has shown significant potential for enhancing drug delivery and improving cancer therapeutics. Tumours can no longer be considered in isolation as a mass of rapidly proliferating neoplastic cells. Instead, tumours are a dynamic network of immune and endothelial cells connected by a chaotic and aberrant vasculature system. High IFP, hypoxia and low pH have now all be shown as indicative of the TME. It is known that these characteristics impede the delivery and efficacy of numerous chemotherapeutics. However, evidence presented within this review suggest that nanoparticle therapies can exploit these aberrant characteristics, actively or passively accumulating within tumour tissue. Consequently, nanoparticle therapies are widely considered to represent the next-generation of cancer therapeutics.

Currently, translation of nanoparticle therapies to clinic remains limited due to poor stability, circulation time and internalisation. Conjugation of stealth molecules often improves stability, however, as discussed stealth molecules can themselves impede target cell uptake. This review outlines a number of possible modifications that could circumvent these difficulties, including the use of cell-penetrating peptides and the development of TME responsive nanoparticles. To date, these approaches remain in their infancy with in vitro validation remaining challenging. Yet, as our knowledge and understanding of these relatively new technologies continue to emerge, it is likely that therapeutic strategies which exploit features of the tumour microenvironment will eventually hold prominence routine in clinical practice.

## Abbreviations

5-FU: 5-fluorouracil
AuNP: gold nanoparticle
BBTB: blood-brain tumour barrier
bFGF: basic fibroblast growth factor
BPs: bisphosphonates
CAF: cancer associated fibroblast
CPP: cell penetrating peptide
DLS: dynamic light scattering
ECM: extracellular membrane
EDN-1: endothelin-1
EPR: enhanced permeability and retention
GPN: gelatin coated nanoparticle
HIF-1α: hypoxia-inducible factor 1 alpha
IFP: interstitial fluid pressure
IC_50_: inhibitory concentration 50%
ICP-MS: inductively coupled mass spectrometry
LOX: lysyl oxidase
MDSC: myeloid-derived suppressor cell
MMP: matrix metalloproteinase
NF-κB: nuclear factor kappa
NK: natural killer cell
PBS: phosphate buffered saline
PDGF: platelet derived growth factor
PEG: poly-ethylene glycol
PGE_1_: Prostaglandin E1
PIM-1: PIM-1 oncogene
RES: reticuloendothelial system
T-REG: T-regulatory Cell
TAM: Tumour Associated Macrophage
TAN: Tumour Associated Neutrophil
TEM: transmission electron microscopy
TME: tumour microenvironment
VEGF: vascular endothelial growth factor
